# Ptf1a triggers GABAergic neuronal cell fates in the retina

**DOI:** 10.1186/1471-213X-7-110

**Published:** 2007-10-02

**Authors:** Jean-Philippe Dullin, Morgane Locker, Mélodie Robach, Kristine A Henningfeld, Karine Parain, Solomon Afelik, Tomas Pieler, Muriel Perron

**Affiliations:** 1 UMR CNRS 8080, Université Paris Sud, Bât. 445, 91405 Orsay, France; 2 DFG-Center of Molecular Physiology of the Brain, Department of Developmental Biochemistry, University of Goettingen, Justus-von-Liebig-Weg 11, 37077 Goettingen, Germany

## Abstract

**Background:**

In recent years, considerable knowledge has been gained on the molecular mechanisms underlying retinal cell fate specification. However, hitherto studies focused primarily on the six major retinal cell classes (five types of neurons of one type of glial cell), and paid little attention to the specification of different neuronal subtypes within the same cell class. In particular, the molecular machinery governing the specification of the two most abundant neurotransmitter phenotypes in the retina, GABAergic and glutamatergic, is largely unknown. In the spinal cord and cerebellum, the transcription factor Ptf1a is essential for GABAergic neuron production. In the mouse retina, Ptf1a has been shown to be involved in horizontal and most amacrine neurons differentiation.

**Results:**

In this study, we examined the distribution of neurotransmitter subtypes following *Ptf1a *gain and loss of function in the Xenopus retina. We found cell-autonomous dramatic switches between GABAergic and glutamatergic neuron production, concomitant with profound defects in the genesis of amacrine and horizontal cells, which are mainly GABAergic. Therefore, we investigated whether Ptf1a promotes the fate of these two cell types or acts directly as a GABAergic subtype determination factor. In ectodermal explant assays, Ptf1a was found to be a potent inducer of the GABAergic subtype. Moreover, clonal analysis in the retina revealed that *Ptf1a *overexpression leads to an increased ratio of GABAergic subtypes among the whole amacrine and horizontal cell population, highlighting its instructive capacity to promote this specific subtype of inhibitory neurons. Finally, we also found that within bipolar cells, which are typically glutamatergic interneurons, Ptf1a is able to trigger a GABAergic fate.

**Conclusion:**

Altogether, our results reveal for the first time in the retina a major player in the GABAergic *versus *glutamatergic cell specification genetic pathway.

## Background

Five classes of retinal neurons (photoreceptors, ganglion, amacrine, horizontal and bipolar cells) and one type of glial cells (Müller cells) are produced in a stereotypical order from a multipotent pool of retinal progenitor cells [1-4]. The conserved order of appearance during development is thought to rely on the progenitor capacity to pass through several intrinsically determined competence states, during which they are capable of giving rise to only a limited subset of cell types under the influence of extrinsic signals [5,6]. Previous studies have established that these distinct retinal cell types are specified through a combinatorial code of bHLH and homeodomain transcription factors [5,7-9]. Importantly, these studies define these transcription factors as cell type determining factors.

Behind the simplicity of the retina, encompassing only six major cell types, lies a large diversity of retinal subtypes forming a complex and subtle structure [10,11]. Mammalian retinas contain approximately 55 distinct subtypes of neurons, based on shape and arborization of these cells. There are many reasons to believe that each of these cell types has a distinct physiological function [10]. Indeed, electrophysiological experiments have thus far revealed specific functions for 22 morphological distinct subtypes. A typical mammalian retina for example, contains ten to fifteen subtypes of retinal ganglion cells and nine to eleven subtypes of cone bipolar cells, based on their synaptic inputs from cone photoreceptors and their sub-laminar localization [10,12]. Amacrine cells, which modulate synaptic activity between bipolar and ganglion cells, constitute the most diverse cell type within the retina [10,11]. In mammals, they can be further classified into 29 different amacrine subtypes based on criteria such as sub-laminar localization (the inner plexiform layer, the ganglion cell layer and the inner part of the inner nuclear layer), morphology (e.g. starburst, parasol or midget) and neurotransmitter type (e.g. GABAergic, glycinergic, dopaminergic or serotoninergic) [11]. In the anuran retina, the number of amacrine subtype cells stands at no less than 21 [13].

The current step-wise model explaining how these multiple subclasses of cells are specified, presages that retinogenic factors first determine cell types, and that another pool of transcription factors subsequently specifies retinal neuronal subtypes [14]. Past work in developmental neurobiology has yielded significant insight into the molecular codes underlying the specification of the six major cell classes. However, little attention has been paid to the mechanisms sustaining the generation of the different neuronal subclasses in the retina. For example, targeted deletion studies have revealed that Vsx1, a paired-like homeodomain factor, and Bhlhb4, an Olig-family bHLH factor, are required for the development of cone bipolar cell and rod bipolar cell subtypes, respectively. Noteworthy, phenotypic analysis revealed that these factors are not involved in the determination of the cellular subtypes but are rather required for their late differentiation [15-17]. Targeted deletion of *Bhlhb5*, an Olig family bHLH factor, causes the loss of gamma-aminobutyric acid (GABA)-producing amacrine and Type 2 OFF-cone bipolar cells. This result, together with genetic interaction studies, argues for a crucial role of Bhlhb5 in the specification of both amacrine and bipolar subtypes [18]. However, the transcription factors responsible for the specification of the majority of retinal subtypes remain to be identified.

For the most part, the molecular cues governing fate determination for the two most abundant neurotransmitter phenotypes in the retina, GABA and glutamate, remain unexplored. Several transcription factors have however been reported as key players in this process within other regions of the central nervous system. For instance, ectopic expression of the homeobox gene *Tlx3 *is sufficient to repress GABAergic differentiation and induce the formation of glutamatergic cells in the spinal cord [19]. On the other hand, the bHLH genes *Mash1 *and *Heslike*, and the homeodomain genes *Nkx2.1*, *Dlx1/2*, and *Gsh1/2 *are involved in the development of GABAergic neurons in the telencephalon or spinal cord [20-24]. Recently, Ptf1a (pancreas transcription factor 1a), a Twist subclass of bHLH transcription factor, has been found to be involved in driving neural precursors to differentiate into GABAergic neurons in the cerebellum and in the dorsal horn of the spinal cord [25-28]. In addition to the spinal cord, hindbrain and cerebellum, Ptf1a is also expressed in the retina during development [29-31]. Two reports studying retinal explants of knock out mice suggest that Ptf1a is involved in the genesis of horizontal and most amacrine neurons, downstream of the forkhead transcription factor Foxn4 [30,31]. Fujitani *et al*. indeed found that inactivation of *Ptf1a *results in a complete loss of horizontal cells, a profound decrease of amacrine cells and an increase in ganglion cells [30]. Nakkai *et al*. found that GABAergic and glycinergic amacrine cells, as well as horizontal cells, were completely missing in Ptf1a-knockout retinal explants [31]. We further explored the retinal-specific roles of Ptf1a, with an emphasis on neurotransmitter phenotypes, through the use of both gain and loss of function analysis in Xenopus embryos. We found dramatic cell-autonomous switches between GABAergic and glutamatergic neuron production, correlated with profound defects in the genesis of amacrine and horizontal cells, which are mainly GABAergic. Therefore, we investigated whether Ptf1a determines the fate of these two cell types or whether it acts directly as a GABAergic subtype determining factor. We found that in animal cap assays Ptf1a is a potent GABAergic subtype inducer factor. In addition, we showed that among a particular cell type, *Ptf1a *overexpression leads to an increased ratio of GABAergic subtype. Together, this study reveals for the first time a key component of GABAergic *versus *glutamatergic cell specification genetic pathway in the retina.

## Results

### *Ptf1a *expression in the retina defines a subpopulation of precursor cells

**Figure 1:**
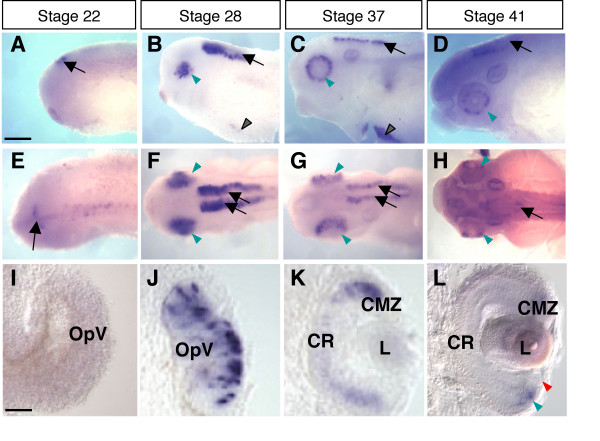
***Ptf1a *is expressed in a subpopulation of retinal precursors**. Lateral (A-D) or dorsal (E-H) views of Xenopus embryos after *Ptf1a *whole-mount *in situ *hybridization at the indicated developmental stages. (A, E) *Ptf1a*-positive cells are detected in the presumptive hindbrain (arrow) at stage 22. (B-D, F-H) At later tailbud stages, *Ptf1a *transcripts are detected in the retina (green arrowhead), the pancreas (black arrowhead) and the hindbrain (arrow). (I-L) As shown on retinal sections, *Ptf1a *starts to be expressed at late optic vesicle stages (I *versus *J), in a subpopulation of retinoblasts (spotty expression pattern in J). (K, L) Its expression progressively vanishes from the central retina as differentiation proceeds and is finally restricted to the CMZ (green arrowhead), although excluded from the stem cell containing region (red arrowhead). OpV: optic vesicle; CMZ: ciliary marginal zone; CR: central retina; L: lens. Scale Bars represents 300 μm (A-H) or 50 μm (I-L).

The spatial and temporal distribution of *Ptf1a *transcripts during Xenopus laevis retinogenesis was analyzed using whole-mount *in situ *hybridization (Fig. 1A–H). *Ptf1a *expression was detected in the optic vesicle from stage 24/25 onwards (not shown). In order to finely characterize *Ptf1a *expression in the neural retina, we sectioned embryos transversely at key stages of retinogenesis. In the proliferating neuroepithelium, *Ptf1a *displayed a patchy expression pattern, with intense staining within discrete groups of cells (Fig. 1J). As retinogenesis proceeds, staining progressively declined in the central retina while remaining high in the margins, suggesting that *Ptf1a *mRNA expression is turned off in differentiating cells and exclusively maintained in precursor cells (Fig. 1K). Accordingly, at stage 41, when all cells in the central retina are post-mitotic, *Ptf1a *expression became restricted to the ciliary marginal zone (CMZ), the only retinal region where retinogenesis is still occurring post-embryonically (Fig. 1L). Noticeably, *Ptf1a*-positive cells were not detected in the most peripheral region of the CMZ, where stem cells reside. Altogether, these data suggest that *Ptf1a *mRNA is restricted to retinal precursors and features a subpopulation of retinoblasts, as inferred by its scattered distribution.

### Pft1a cell-autonomously promotes amacrine and horizontal cell genesis

**Figure 2:**
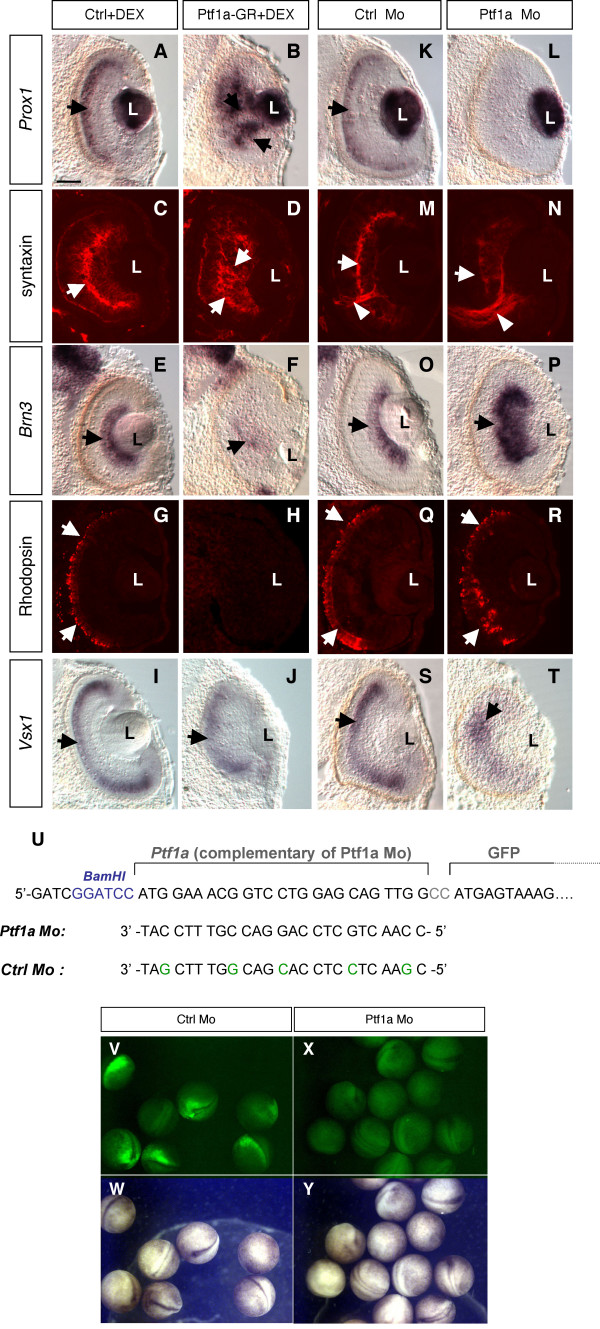
**Ptf1a is required for proper amacrine and horizontal cell genesis**. (A-T) *In situ *hybridization (A-B, E-F, I-J, K-L, O-P, S-T) or immunofluorescence (C-D, G-H, M-N, Q-R) analysis of cell-type specific marker expression in stage 39/40 retinas, following *Ptf1a-GR *or *Ptf1a *Mo injection in two cell stage embryos. (A-J) *Prox1 *(horizontal marker, arrows in A and B) and syntaxin (optic nerve and inner plexiform layer marker, arrows in C and D) expressions are expanded in the central retina following *Ptf1a *overexpression. In contrast, both *Brn3 *(ganglion cell marker, arrows in E and F) and rhodopsin (photoreceptor marker, arrows in G, H) display highly diminished expression. Expression of *Vsx1 *(bipolar marker, arrows in I and J) does not show any clear decrease although significant disorganization is occasionnaly observed in severe phenotypes (not shown). (K-T) Compared to control ones, *Ptf1a *Mo injected retinas display virtual absence of *Prox1 *expression (arrows in K, L) and reduced syntaxin staining in the IPL (arrows in M and N). Note the increased size of the optic nerve in Ptf1a knocked down retinas (arrowheads in M and N) that is consistent with the increased expression of the ganglion cell marker *Brn3 *(arrows in O and P). Both rhodopsin (arrows in Q and R) and *Vsx1 *(arrows in S and T) appear normally expressed albeit with a disorganized expression pattern. Ctrl: control; L: lens. Scale Bar represents 50 μm. (U-Y) Test of Ptf1a Morpholino efficiency. *In vivo *GFP fluorescence was analysed following co-injection of either *Ptf1a *Mo or Ctrl Mo with a chimeric *GFP *construct fused downstream *Ptf1a *Mo complementary region. (U) Schematic representation of the construct and sequences of Morpholino oligonucleotides. (V-Y) GFP expression can be observed in Ctrl Mo injected neurulas, while it is efficiently inhibited in *Ptf1a *Mo injected ones.

The *Ptf1a *expression profile led us to analyze whether it might function in the context of Xenopus retinogenesis. We first injected an inducible form of *Ptf1a *mRNA (*Ptf1a-GR*) into two-cell stage embryos. Protein activity was induced at stage 22 by addition of dexamethasone (DEX), and effects on retinal cell type genesis were monitored using specific markers. *Ptf1a *overexpressing retinas displayed substantial perturbations in laminar organization. Expression of the horizontal marker *Prox1 *was strongly increased following *Ptf1a *overexpression (Fig. 2A,B). Syntaxin staining of the inner plexiform layer (IPL) was enhanced as well, suggesting a greater density of amacrine cells (Fig. 2C,D). In contrast, highly reduced *Brn3 *expression (Fig. 2E,F) and virtual absence of rhodopsin staining (Fig. 2G,H) indicated that ganglion cells and photoreceptors were profoundly missing. Expression of the bipolar marker *Vsx1*, albeit disorganized, did not reveal any evident reduction (Fig. 2I,J). Since *Vsx1 *is also expressed in retinal precursors, the possibility remained that *Vsx1*-positive cells could be undifferentiated precursors dispersed in the central retina. However, this was excluded, as the expression of the precursor marker *Xath5*, was correctly restricted to the CMZ (data not shown). Finally, cell death was examined at stage 30, 35 and 39. The number of apoptotic cells in *Ptf1a *overexpressing retinas did not differ from that of control embryos at any stage tested (data not shown). Thus, Ptf1a gain of function drastically augments horizontal and amacrine cell production at the expense of photoreceptor and ganglion cells.

Parallel loss of function experiments were then performed using an antisense Morpholino oligonucleotide designed against the ATG-containing 5' end of *Ptf1a *(*Ptf1a *Mo; Fig. 2U–Y). Inhibition of Ptf1a resulted in complete loss of *Prox1*-positive horizontal cells (Fig. 2K,L) and severe decrease of syntaxin-stained amacrine fibers (Fig. 2M,N), along with strong increase of the ganglion cell marker *Brn3 *expression (Fig. 2O,P). Despite the significant laminar disorganization, rhodopsin and *Vsx1 *stainings revealed an apparent normal generation of photoreceptor and bipolar cells, respectively (Fig. 2Q–T). In contrast to the gain of function experiment, apoptosis was significantly enhanced upon Ptf1a inhibition during retinogenesis (data not shown). These results indicate that at least part of the *Ptf1a *knocked-down retinal precursors either undergo apoptosis or are biased towards a ganglion fate at the expense of amacrine and horizontal cell fates, similarly to previous findings in mouse retina [30,31].

**Figure 3:**
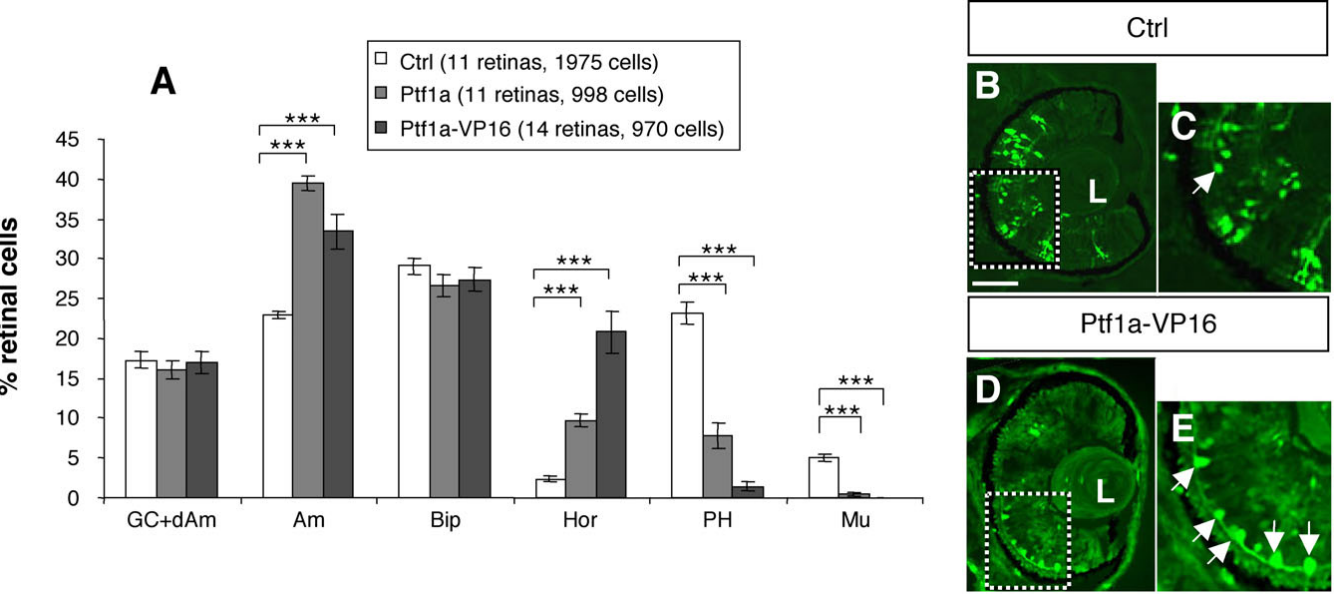
***Ptf1a *cell autonomously biases retinal precursors towards amacrine and horizontal cell fates**. Analysis of cell types distribution in stage 40/41 retinas, following *Ptf1a *or *Ptf1a-VP16 *lipofection. (A) Percentage of retinal cell types. Values are given as mean +/- s.e.m. p < 0.001 (***) (Student's t test). (B-E) Typical sections of retinas transfected with *GFP *alone (B, C) or *GFP *plus *Ptf1a-VP16 *(D, E), showing the dramatic increase of horizontal cells (arrows). C and E are higher magnifications of the dotted square delineated regions in B and D. Ctrl: control; L: lens; GC: ganglion cells; dAm: displaced amacrine cells; Bip: bipolar cells; Hor: horizontal cells; PH: photoreceptors; Mu: Müller cells. Scale Bar represents 50 μm.

The above data suggest that Ptf1a is required for proper generation of amacrine and horizontal cells. In order to investigate whether Ptf1a acts cell autonomously, *Ptf1a *or a *Ptf1a *chimeric construct fused to the VP16 transactivation domain (*Ptf1a-VP16*) were overexpressed in the developing retina by *in vivo *lipofection at stage 17/18. GFP expressing plasmid was used as a tracer, allowing for the identification of transfected cells in stage 40/41 embryos, when most cells in the central retina are fully differentiated [1]. Both *Ptf1a *constructs led to very similar changes in cell type distribution, although *Ptf1a-VP16 *phenotype was clearly enhanced (Fig. 3A). In agreement with two-cell stage injection experiments, *Ptf1a *overexpressing clones exhibited increased proportions of horizontal and amacrine cells compared to control ones (Fig. 3A–E). This was, as expected, at the expense of photoreceptors and ganglion cells. Of note, although the global proportion of cells in the ganglion cell layer was not affected, ganglion cells were significantly depleted, whereas displaced amacrine cells were drastically increased (see below and Fig. 5D,K–P). Moreover, Müller glial cells were virtually absent among *Ptf1a-VP16 *lipofected clones. Altogether, these data suggest that Ptf1a acts cell autonomously to bias precursor cells towards amacrine and horizontal destinies.

### *Ptf1a *promotes a GABAergic cell fate of retinal precursor cells

**Figure 4:**
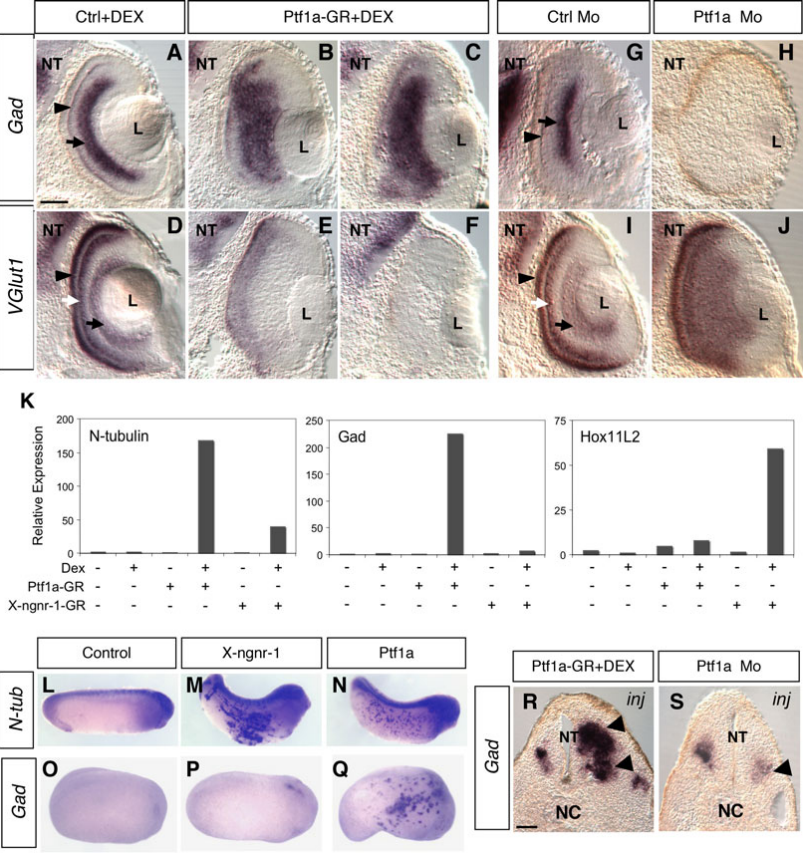
**Ptf1a misexpression dramatically affects the ratio of GABAergic *versus *glutamatergic neurons**. (A-J) *In situ *hybridization analysis of glutamic acid decarboxylase (*Gad*) and glutamate transporter 1 (*Vglut1*) in stage 39/40 retinas, following *Ptf1a-GR *or *Ptf1a *Mo injection in two-cell stage embryos. (A-F) *Ptf1a *overexpression results in a drastic increase of *Gad *staining (A-C) at the expense of *VGlut1 *staining (D-F). Shown in C and F are strong phenotypes compared to milder ones in B and E. (G-J) Conversely, Ptf1a knocked-down retinas display virtual absence of *Gad *expression (G, H), while that of *VGlut1 *is highly expanded (I, J). Arrow and arrowhead in A and G point to *Gad *expression domain in amacrine and horizontal layers, respectively. Black arrow, white arrow and black arrowhead in D and I point to *VGlut1 *expression domain in ganglion, bipolar and photoreceptor cells, respectively. Ctrl: control; L: lens; NT: neural tube. Scale Bar represents 50 μm. (K) Real-time RT-PCR analysis of *Gad *and *Hox11L2 *expression in animal cap assays (equivalent stage 16), following *X-ngnr-1-GR *(25 pg) or *Ptf1a-GR *(50 pg) overexpression. *N-tubulin *was used as a control for X-ngnr-1 and Ptf1a neuralizing activities. Expression levels were normalized to *ornithine decarboxylase *(*ODC*). The expression levels were measured using a standard curve for each analyzed gene. All measurements were done in duplicates and the values in the figures represent the mean of a representative experiment. (L-Q) *In situ *hybridization analysis of *N-tubulin *(*N-tub*) and *Gad *expression in whole embryos (stage 28 in L-N and 24 in O-Q), following *X-ngnr-1 *or *Ptf1a *mRNA injection in one of two blastomere of two-cell stage embryos. (R, S) *In situ *hybridization analysis of *Gad *expression in the neural tube (stage 28), following *Ptf1a-GR *or *Ptf1a *Mo injection in one of two blastomere of two-cell stage embryos. The *Ptf1a-GR *injected embryos were induced with dexamethasone at stage 11. NT: neural tube; NC: notocord; inj: injected side. Scale Bar represents 100 μm.

Amacrine and horizontal interneurons have been described in different model organisms as the two major GABAergic populations in the retina, whereas photoreceptor, ganglion and bipolar cells are considered as mainly glutamatergic [32]. As recent studies have highlighted a role for Ptf1a in the specification of GABAergic neurons in both the murine spinal cord [25] and cerebellum [26], we wondered whether Ptf1a might have a similar function in the retina. As expected, expression of glutamic acid decarboxylase (*Gad*), the rate limiting enzyme for GABA biosynthesis, was restricted to horizontal and amacrine cell layers (Fig. 4A,G). Conversely, expression of *VGlut1*, which encodes a glutamate transporter, could be detected in photoreceptors, bipolar and ganglion cell layers (Fig. 4D,I). *Ptf1a *overexpression resulted in dramatic increase of *Gad *staining in the retina (Fig. 4A–C) at the expense of *VGlut1 *expression (Fig. 4D–F). In contrast, Ptf1a knocked-down retinas exhibited severe to complete abrogation of *Gad*-expressing cells (Fig. 4G,H), with concomitant enlargement of the *VGlut1*-expressing domain (Fig. 4I,J).

Such phenotypes are consistent with the above cell type distribution data and thus raise the question of whether Ptf1a determines the fate of cell types that are mainly GABAergic or whether it directly acts as a GABAergic subtype determination factor. To further explore this second hypothesis, we tested the capacity of Ptf1a to activate *Gad *expression in ectodermal explants (animal caps). Similar to X-ngnr-1, Ptf1a was able to induce neurogenesis in naive animal caps, as shown by *N-tubulin *expression. Importantly, overexpression of *Ptf1a*, but not that of *X-ngnr-1*, resulted in a significant induction of *Gad *expression (Fig. 4K). On the other hand, X-ngnr-1 but not Ptf1a, strongly activated the expression of *XHox11L2/Tlx3*, a selector gene determining glutamatergic over GABAergic cell fates in the spinal cord [19] (Fig. 4K). In whole embryos, Ptf1a still behaved as a proneural gene, inducing, as *X-ngnr-1*, ectopic *N-tubulin *expression in the epidermis (Fig. 4L–N). However, only Ptf1a was able to drive *Gad *ectopic expression (Fig. 4O–Q). Finally, in the neural tube, the *Gad *expression domain was substantially extended in *Ptf1a*-injected embryos (Fig. 4R). Conversely, injection of *Ptf1a *Mo strongly reduced *Gad *expression in the hindbrain (Fig. 4S). These results demonstrate that Ptf1a can act as a GABAergic subtype inducing factor and suggest that it is specifically required in the retina for GABAergic cell genesis at the expense of glutamatergic neurons.

### Ptf1a differentially affects subtypes of amacrine and horizontal neurons

**Figure 5:**
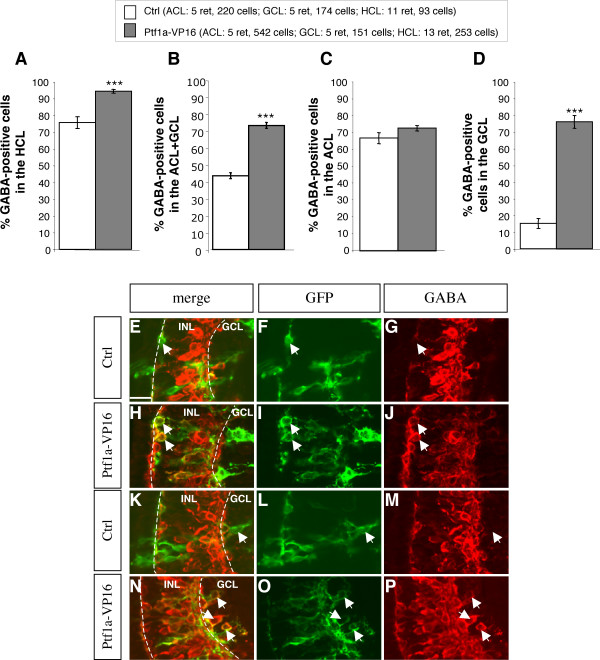
**Ptf1a overexpression leads to enhanced proportions of GABAergic horizontal and displaced amacrine cells**. Analysis of GABAergic cell proportions among GFP-positive horizontal and amacrine transfected cells in stage 40/41 retinas, following *Ptf1a-VP16 *lipofection. (A-D) Quantification of GABA-positive cells in the horizontal, amacrine and ganglion cell layers as indicated. Values are given as mean +/- s.e.m. p < 0.001 (***) (binomial test). (E-P) Typical sections of retinas lipofected with *gap-GFP *alone (E-G, K-M) or *gap-GFP *plus *Ptf1a-VP16 *(H-J, N-P), immunostained with an anti-GABA antibody. Arrows in E-G and K-M point to examples of *gap-GFP*-transfected GABA-negative horizontal and ganglion cell, respectively, in control retinas. Arrows in H-J and N-P point to GABA-positive horizontal and displaced amacrine cells, respectively, in *gap-GFP *plus *Ptf1a-VP16 *lipofected retinas. Ctrl: control; HCL: horizontal cell layer; ACL: amacrine cell layer; GCL: ganglion cell layer; INL: inner nuclear layer. Scale Bar represents 30 μm.

In order to demonstrate that Ptf1a does not simply specify amacrine and horizontal cell fates but rather specifically pushes retinal precursors to adopt a GABAergic phenotype, we analyzed the proportion of GABA-positive cells within horizontal and amacrine *Ptf1a*-overexpressing cells, through a clonal analysis. We found that the ratio of GABAergic neurons in both cell types was largely increased compared to controls (Fig. 5A–P). Noticeably, regarding amacrine cells, this increase was essentially due to a dramatically enhanced proportion of GABA-positive displaced amacrine cells (Fig. 5B–D,K–P). This result suggests that *Ptf1a *has the particular function of differentially favoring GABAergic subtypes among amacrine and horizontal cells.

**Figure 6:**
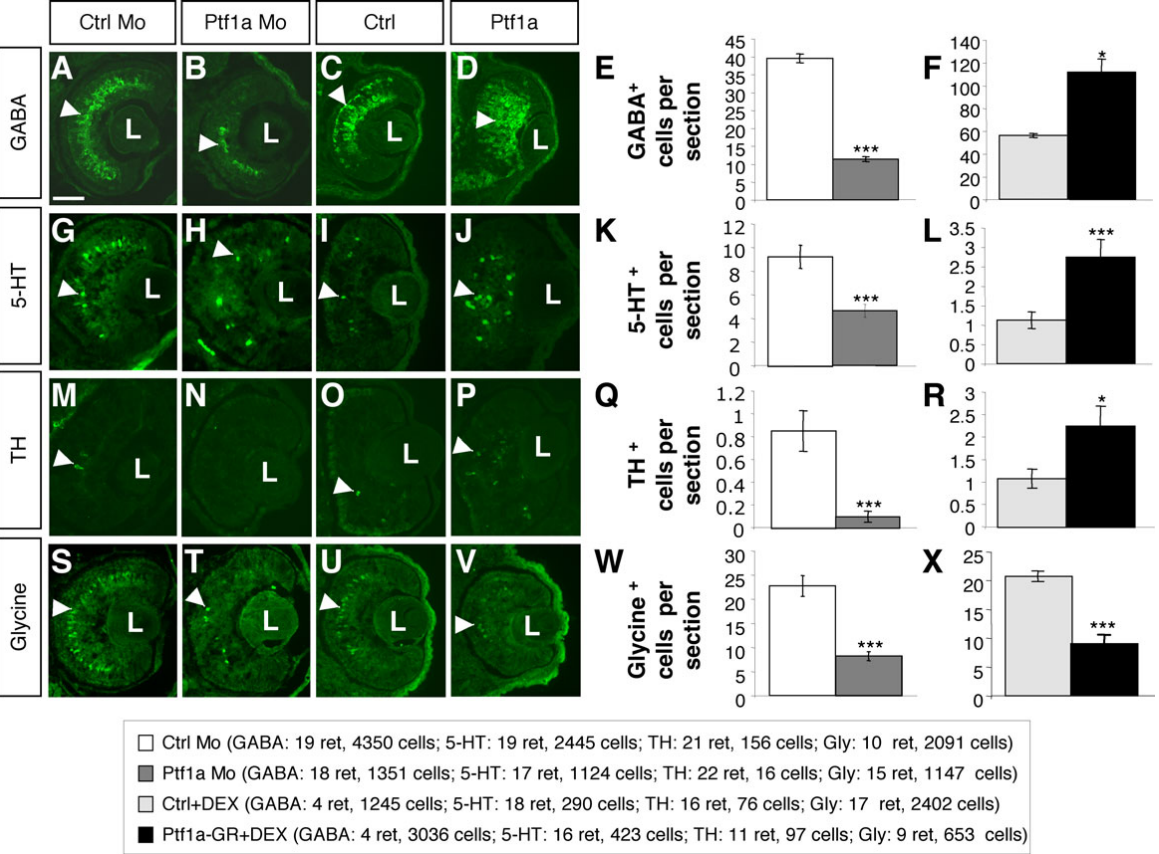
***Ptf1a *misexpression differentially affects amacrine cell subtypes genesis**. Quantification of GABA-, serotonin (5-HT)-, tyrosine hydroxylase (TH)- and glycine-positive cells, following *Ptf1a *Mo (stage 40/41) or *Ptf1a-GR *(stage 39, except in O, P, R stage 41) injection in two cell stage embryos. (A-D, G-J, M-P, S-V) Typical sections of control, Ptf1a knocked-down or *Ptf1a *overexpressing retinas, immunostained for GABA, 5-HT, TH or glycine (arrows) as indicated. Graphs indicate the average numbers of GABA-, 5-TH-, TH- or glycine-positive cells per retinal section in each condition. Values are given as mean +/- s.e.m. p < 0.001 (***), p < 0.05 (*) (Student's t test). Ctrl: control; L: lens. Scale Bar represents 50 μm.

We next examined whether other neurotransmitter subtypes of amacrine cells were affected following *Ptf1a *misexpression. Injection experiments revealed that the number of serotoninergic (serotonin-immunoreactive) and dopaminergic (tyrosine hydroxylase-immunoreactive) amacrine cells were, like GABAergic ones, decreased following *Ptf1a *loss of function and increased upon overexpression (Fig. 6A–R). We also found that the number of glycine-positive cells was significantly lower in the retina of *Ptf1a *Mo-injected embryos (Fig. 6S,T,W). Surprisingly, however, in *Ptf1a *overexpressing retinas, a severe decrease of glycinergic amacrine cells could be observed (Fig. 6U,V,X). To rule out potential secondary effects resulting from impaired retinal morphogenesis, we turned to clonal analysis through *in vivo *lipofection. In contrast to the *Ptf1a *two-cell stage injection data, *Ptf1a-VP16 *overexpressing clones displayed a significant increase in the proportion of glycine-positive amacrine cells (50% n = 4 retinas, 176 cells *versus *33% n = 5 retinas, 185 cells; p < 0.001). Thus, Ptf1a seems to be able, at least in some conditions, to favor glycinergic, dopaminergic, and serotonin-positive cell genesis, at the expense of yet unindentified other amacrine subtypes.

### Ptf1a converts glutamatergic bipolar interneurons into GABAergic cells

**Figure 7:**
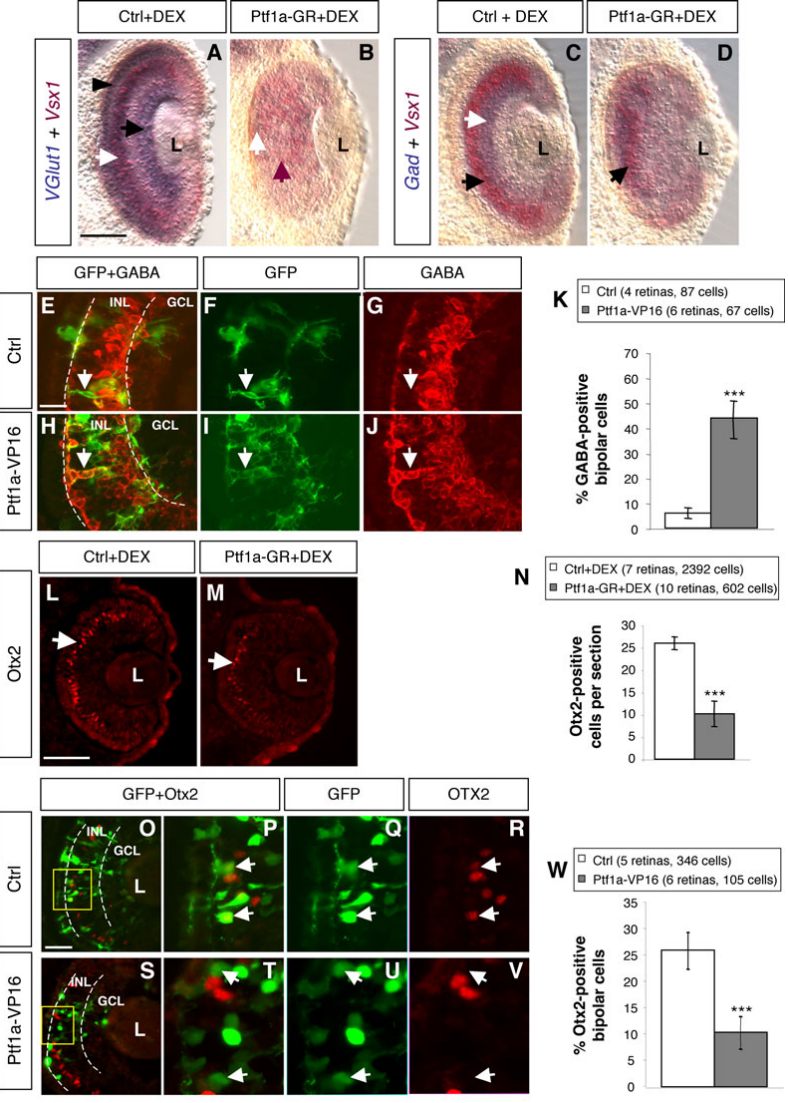
***Ptf1a *overexpression triggers the conversion of glutamatergic bipolar interneurons into GABAergic cells**. (A-D) Double *in situ *hybridization analysis of *Vsx1/VGlut1 *or *Vsx1/Gad *co-expression (stage 39/40), following *Ptf1a-GR *injection in both blastomeres of two cell stage embryos. (A) In control retinas, *VGlut1 *stains ganglion (black arrow) and photoreceptor cells (black arrowhead) and colocalizes with *Vsx1 *in bipolar cells (white arrow). (B) Double *VGlut1*/*Vsx1 *staining is highly reduced in *Ptf1a *overexpressing retinas (white arrow: region of double staining persistence, red arrow: *Vsx1*-positive region with no *VGlut1 *staining). (C) *Gad *(white arrow) and *Vsx1 *(black arrow) have exclusive expression patterns in control retinas. (D) Upon *Ptf1a *overexpression, regions of double *Gad*/*Vsx1 *staining become apparent (arrow). (E-K) Analysis of GABAergic bipolar cell proportion in stage 40/41 retinas, following *Ptf1a-VP16 *lipofection. (E-J) Typical sections of retinas transfected with *gap-GFP *alone (E-G) or *gap-GFP *plus *Ptf1a-VP16 *(H-J), immunostained with an anti-GABA antibody. Arrows point to a transfected GABA-negative bipolar cell in a control retina (E-G) and to a GABA-positive one in *Ptf1a-VP16 *overexpressing retina (H-J). (K) Quantification of GABA-positive bipolar cells among transfected cells. (L-N) Immunofluorescence analysis of *Otx2 *expression (stage 39), following *Ptf1a-GR *injection in one of two blastomere of two cell stage embryos. (L, M) Typical sections of control (L) and *Ptf1a *overexpressing (M) retinas immunostained for Otx2 (arrow). (N) Quantification of Otx2-positive cells per retinal section in each condition. (O-W) Analysis of Otx2-positive bipolar cell proportion in stage 40/41 retinas, following *Ptf1a-VP16 *lipofection. (O, V) Typical sections of retinas transfected with *GFP *alone (O-R) or *GFP *plus *Ptf1a-VP16 *(S-V), immunostained with an anti-Otx2 antibody. (P-R) and (T-V) are higher magnifications of the delineated regions in O and S, respectively. Arrows point to transfected Otx2-positive bipolar cells in a control retina (P-R) and Otx2-negative ones in *Ptf1a-VP16 *overexpressing retina (P-R). (W) Quantification of Otx2-positive bipolar cells among transfected cells. Values are given as mean +/- s.e.m. p < 0.001 (***)(Student's t test in N, binomial test in K and W). Ctrl: control; L: lens; INL: inner nuclear layer; GCL: ganglion cell layer. Scale Bar represents 50 μm in A-D, L, M and 30 in E-J, O-V.

In contrast to glutamatergic photoreceptors and ganglion cells that are extensively suppressed upon *Ptf1a *overexpression, bipolar cells appear largely unaffected both in two-cell stage injection and lipofection experiments (Fig. 1I–J and Fig. 3A). Regarding the global reduction of *VGlut1 *staining in *Ptf1a-GR *injected retinas (Fig. 4D–F), it is likely that a significant proportion of bipolar cells must have altered its fate towards another neurotransmitter phenotype. The existence, in physiological conditions, of a minority GABAergic bipolar subpopulation in amphibians [33,34] led us to postulate that Ptf1a gain of function might drive bipolar precursors to acquire a GABAergic phenotype. As a first attempt to test this hypothesis, we performed double *in situ *hybridizations using the *Vsx1 *bipolar probe in combination with either the *VGlut1 *or *Gad *probes. As expected, *Vsx1 *staining appears to be co-localized with the *VGlut1*-positive layer of the INL but not with the *Gad*-positive one in control embryos (Fig. 7A,C). In contrast, in *Ptf1a *overexpressing retinas, double *Vsx1*/*Vglut1 *staining was highly reduced (Fig. 7B), while regions co-expressing both *Vsx1 *and *Gad *were apparent (Fig. 7D), suggesting that some glutamatergic bipolar cells transfated towards a GABAergic phenotype. This result could be further assessed through clonal lipofection analysis (Fig. 7E–K). Indeed, we observed a dramatic increase in the proportion of GABA-positive cells among *Ptf1a*-lipofected cells localized in the bipolar cell layer compared to control ones (43% *versus *5%).

It has recently been shown that the Otx2 transcription factor, which is selectively expressed in a subpopulation of bipolar cells [35], controls identity and fate of glutamatergic progenitors of the thalamus by repressing the alternative GABAergic differentiation program [36]. We thus wondered whether the capacity of Ptf1a to force GABAergic fate of bipolar cells might be related to effects on *Otx2 *expression. *Ptf1a-GR *injection in two-cell stage embryos indeed led to a drastic reduction of *Otx2*-positive cells in the outer portion of the INL (Fig. 7L–N). Consistently, clonal analysis revealed a decreased proportion of *Otx2*-positive cells among *Ptf1a*-overexpressing bipolar cells compared to a control experiment (7% *versus *27%, Fig. 7O–W). Thus, Ptf1a overexpression leads to an unbalance between GABA and *Otx2*-positive bipolar cell subtypes.

## Discussion

Many transcription factors affect the diversity and numbers of distinct retinal cell types. In contrast, studies identifying molecular cues underlying retinal subtype specification are limited. We focused our attention on GABAergic inhibitory neurons and glutamatergic excitatory neurons. In the central nervous system, a balance of excitation and inhibition is essential for nearly all functions, and imbalances can result in sensory disorders. In the retina, the complex network of excitatory and inhibitory pathways contributes to edge sharpening, contrast enhancement, spatial summation, noise averaging, and other forms of signal processing. Thus far, the molecular players governing the determination of glutamatergic and GABAergic neurons in the retina have surprisingly remained unexplored. We report here on the key role of Ptf1a in this process. To reach this conclusion, the role of Ptf1a during retinogenesis was examined using both gain and loss of function approaches through different experimental strategies, including histological assessment of retinal cell types and subtypes, clonal analysis and animal cap assays.

The role of Ptf1a in the retina has been studied through its inactivation in mice and analyzed in retinal explants [30,31]; it led to a complete loss of horizontal cells and to a profound decrease of amacrine cells, suggesting that Ptf1a plays a central role in directing retinal progenitors towards these cell fates. In our loss of function analyses in the Xenopus retina, we confirmed a severe reduction of amacrine and horizontal cells. As reported in *Ptf1a*^-/- ^retinal explants, we also observed a concomitant increase of ganglion cells. Our gain of function results reinforce the idea that Ptf1a is able to bias retinal precursors towards amacrine and horizontal fates. However, Nakkai et al. found that in the *Ptf1a *null retina a small number of amacrine precursor cells differentiated to amacrine cells and thus proposed that Ptf1a may contribute to the specification of amacrine cell subtypes rather than to the generation of whole amacrine cells [31]. Indeed, as discussed below, our detailed analysis of the neuronal subtype distribution in both gain and loss of function experiments, revealed that Ptf1a may play a role in neurotransmitter subtype specification rather than simply specifying amacrine and horizontal cell fates.

Past work has identified *Ptf1a *as the responsible gene for permanent neonatal diabetes mellitus associated with pancreatic and cerebellar agenesis [37]. The function of Ptf1a in the cerebellum has been investigated in cerebelless mutant mice and revealed its role in defining GABAergic neuronal fates [26]. In addition, involvement of Ptf1a in the genetic cascade specifying GABAergic over glutamatergic neurons has been established in the dorsal spinal cord [25]. Our GABA and glutamate analysis support the hypothesis that Ptf1a also promotes a switch between GABAergic and glutamatergic fates in the retina. Through clonal analysis, we further demonstrated the instructive capacity of Ptf1a to trigger GABAergic neuron production within amacrine and horizontal cell populations. We thus propose that Ptf1a does not simply determine horizontal and amacrine interneurons but preferentially promotes formation of the GABAergic subtypes of these cells. It is likely that Ptf1a truly acts as a determining factor rather than a differentiation factor, as we found that it is sufficient to drive neuronal differentiation in both ectodermal explants and whole embryos and has the ability to change precursor fate in the retina.

Noticeably, we also found that serotoninergic and dopaminergic amacrine cells, two minority subtypes, were increased upon *Ptf1a *overexpression and decreased upon *Ptf1a *loss of function. Ptf1a may be directly required for the specification of these subtypes. Importantly, however, evidence for a dual expression of GABA and serotonin or GABA and dopamine has been reported in the retina of several species including Xenopus [38-41]. Hence, an alternative hypothesis is that Ptf1a-induced changes in the number of GABAergic cells account for the observed effects on the amount of 5-HT- and TH-positive neurons. As observed in mice [30,31], we also found that glycinergic amacrine cell genesis was inhibited upon Ptf1a loss of function. However, a potential role of Ptf1a in determining this cell subtype remains unclear regarding our gain of function experiments. Indeed, we obtained opposite phenotypes when *Ptf1a *was either overexpressed following retinoblast *in vivo *lipofection (increase) or by two-cell stage mRNA injections (decrease). Such a discrepancy may arise from differences in dose and/or timing of *Ptf1a *transgene expression. As feedback loops are known to regulate amacrine cell genesis [42], the possibility also remains that the dramatic perturbation of the GABAergic cell population, as observed in the two cell stage injection experiments, may non cell-autonomously affect glycinergic cell production.

Using retrograde neuronal tracer experiments, it has been established that some ganglion cells in the outer half of the ganglion cell layer are GABA-positive [43]. We thus wondered whether some of the numerous GABAergic cells found in the ganglion cell layer upon *Ptf1a *overexpression might actually be ganglion cells rather than displaced amacrine cells. Our GAP-GFP (a membrane bound GFP allowing to better stain neuronal fibers) staining highlighted that a few of these cells have processes resembling ganglion cell axons (data not shown), while displaced amacrine cells have neurites oriented towards the INL [44]. Thus, although this situation remains to be quantified with a more accurate technical approach, we suspect that Ptf1a, in addition to increase the proportion of GABAergic displaced amacrine cells in the ganglion cell layer, may also increase the proportion of GABA-positive ganglion cells.

Even though retinal bipolar neurons mainly release the excitatory transmitter glutamate, evidence that certain bipolar cells contain GABA and express *Gad *has been reported in both mammalian and amphibian retinas [33,34,45,46]. Both our double *in situ *experiments and clonal analysis revealed that, in a context of *Ptf1a *overexpression, a considerable proportion of bipolar neurons transfate towards a GABAergic phenotype. Genetic lineage tracing in mice did not reveal that some Ptf1a-expressing precursors are dedicated to bipolar cell production. However, the low number of GABAergic bipolar neurons in wild type retinas may have hindered the identification of a Ptf1a-positive bipolar lineage. Therefore, although this deserves further investigation, our data suggest that Ptf1a may have a role in specifying the GABAergic bipolar subtype. Altogether, these findings indicate that Ptf1a can act as a neuronal subtype determination factor, which instructs neural precursors to differentiate into GABAergic neurons. Our findings thus suggest that Ptf1a constitutes the first identified member of the genetic cascade responsible for GABAergic cell specification in the retina.

Several *in vitro *studies suggest that, in the optic vesicle, some retinal progenitors are not multipotent but are instead differentially biased towards specific cell types, such as rod and bipolar cells or amacrine and horizontal cells for instance [42,47-49]. Molecular identification of these precursor subpopulations is largely lacking. The transcription factor FoxN4 is expressed in a restricted subset of retinal precursors and presumably confers the competence to form amacrine and horizontal cells [50]. *Ptf1a *also labels a subpopulation of retinoblasts at both the levels of mRNA (this study) and protein expression [30,31]. Our data suggest that *Ptf1a*-expressing precursors are biased towards a GABAergic fate. As we showed that *Ptf1a *mRNA starts to be detectable in discrete groups of proliferating precursors as soon as the optic vesicle forms, it is likely that the segregation of a population fated to the GABAergic subtype occurs early in retinal development. This does not fit with the step-wise model supporting the view that subtype specification follows cell class determination. For instance, in the mouse retina it has been proposed that pan-bipolar cells are first specified by Chx10, Math3 and Mash1 and that specification of OFF and ON cone subtypes takes place subsequently under the influence of another set of transcription factors [15]. However, our hypothesis that GABAergic fate specification occurs early in retinogenesis is substantiated by several lineage and transplantation experiments demonstrating that some blastomeres are intrinsically biased, as early as during the early cleavage stage, to produce subsets of neurotransmitter subtypes of amacrine cells [49,51]. Moreover, it has recently been reported that *Rx1 *and *Pax6 *misexpression at the eye field stage differentially affects amacrine subtypes proportions, suggesting that these interneurons are not all specified by a single genetic program [52]. Together with our present work, these findings support the hypothesis that at least some neurotransmitter subtypes are specified by a combinatorial code of transcription factors during early eye development. Importantly, we were able to highlight a role of Ptf1a in GABAergic neuron specification as we analyzed various subtypes of retinal cells. In this regard, it is likely that other transcription factors, described so far as cell type inducers, may actually have a role in the formation of different retinal neurotransmitter subtypes. It would therefore be important to re-evaluate the effects of key components of retinal cell fate specification in the context of cell subtype determination. Such future work would be critical to understand the molecular events that generate the myriad of different cell subtypes in the retina.

## Conclusion

Altogether, our gain and loss of function data identify Ptf1a as a determining factor for retinal GABAergic subtypes neurons as opposed to a determining factor for cell types. Further work examining neurotransmitter cell subtypes should expand our knowledge about retinogenesis, and permit in particular to uncover the genetic network sustaining GABAergic/glutamatergic determination in the retina.

## Methods

### Constructs and Morpholinos

*pCS2+-X-ngnr-1*, *pCS2+-X-ngnr-1-GR *[53], *pCS2+-Ptf1a *and *pCS2+-Ptf1a-GR *[29] have previously been described. *X-ngnr-1-GR *and *Ptf1a-GR *encode glucocorticoid inducible chimeric morphants. Protein activity was induced with 4 μg/ml dexamethasone (DEX, Sigma) in the embryo medium. The *pCS2+-Ptf1a-VP16 *encodes a Ptf1a variant where the VP16 transactivation domain is fused to the carboxylterminus of Ptf1a. The Ptf1a (*Ptf1a *Mo; [29]) and control (Ctrl Mo, 5 mismatches; Fig. 2U) Morpholino (Mo) oligonucleotides were purchased from GeneTools (LLC). Two types of Mo were used: crude Mo (for blastomere injection experiments), and "Special Delivery" Mo, where the non-ionic crude Morpholinos are paired to a complementary carrier DNA (for lipofection experiments) [54,55]. The region complementary to the *Ptf1a *Mo (encompassing 5' untranslated and N-terminal-coding regions of *Ptf1a*) was amplified by PCR from pBK-CMV Ptf1a and cloned in frame downstream of the *GFP *coding sequence in pCS2 plasmid (*pCS2+-Ptf1a(5')-GFP*; Fig. 2U).

### Embryos, microinjections and animal cap explants

Xenopus laevis embryos were obtained by hormone-induced egg laying and *in vitro *fertilization by conventional methods. Capped sense RNAs were prepared from CS2 plasmids after NotI digestion and transcribed using the mMessage mMachine™ *SP6 *kit (Ambion). RNAs were injected in a volume of 5 nl at a concentration of 100–150 pg/nl into two of two or one of two blastomeres of embryos at the two-cell stage. *GFP *RNA (100 pg) co-injection was used to visualize injected cells. Morpholino (Mo) injections were performed as indicated by the manufacturer (GeneTools, LLC). Animal cap explants and real-time RT-PCR analysis were performed as previously described [56]. The following primers pairs were used: ODC [57]; N-tubulin [58]; Gad for: ATGGGCGTCTTACTCCAATG, rev: ATGTCTACATGGCGACCACCACA; Hox11L2 for: GCCAACAAGTACAAGTGCACAG, rev: CAGGAGCCAGACTCACATTGAC.

### *In vivo *lipofection

*pCS2-GFP*, *pCS2-Ptf1a *and *pCS2-Ptf1a-VP16 *were transfected at stage 18 into the presumptive region of the retina as previously described [55,59]. Embryos were fixed at stage 40/41 and cryostat sectioned (12 μm). GFP-positive cells were counted and cell types were identified based upon their laminar position and morphology [60]. In some cases, the *gap-GFP *plasmid (a gift from E. Amaya), which encodes a membrane bound GFP, was used to improve neuronal fiber staining in order to better assess retinal cell type identity.

### Immunohistochemistry and Tunnel assay

Immunohistochemistry was performed as described previously [61], using rabbit polyclonal or mouse monoclonal anti-GFP (Molecular Probe), rabbit polyclonal anti-GABA (ImmunoStar), rabbit polyclonal anti-Glycine (ImmunoSolution), rabbit polyclonal anti-serotonin (Immunostar), mouse monoclonal anti-tyrosine hydroxylase (Immunostar), mouse monoclonal anti-syntaxin (Sigma), rabbit polyclonal anti-Otx2 [35], mouse monoclonal anti-rhodopsin (clone R2–12, a gift from N. Colley), and anti-mouse or anti-rabbit fluorescent secondary antibodies (Alexa, Molecular Probes). Cell nuclei were counterstained with Hoechst (Sigma). Detection of cell apoptosis was carried out with the DeadEnd fluorometric TUNEL system (Promega). Fluorescent stainings were visualized with a Leica HBO100 microscope. Images were then captured using a QICAM camera (QIMAGING) and processed with Adobe Photoshop 7.0 software. Shown in figures are representative data from one experiment that has been performed at least in duplicate.

### *In situ *hybridization

Digoxigenin- or fluorescein-labelled antisense RNA probes, *Ptf1a *[29], *Gad *[62], *XVGlut1 *[62], *XVsx1 * [63], *Brn3.0 * [64], *Prox1 *(a gift from F. Cremisi) and *Xath5 * [65] were generated according to the manufacturer's instructions (Roche). Whole mount *in situ *hybridization was carried out as previously described [66], with an additional step of bleaching just before the proteinase K treatment [67]. For double *in situ *hybridization, *XVsx1 *fluorescein-labeled probes were first revealed with Fast red substrate (Roche). Embryos were then treated in 0.1 M Glycine-HCl pH 2.2 for 10 minutes and processed for *Gad or XVGlut1 *digoxigenin-labeled probe revelation using NBT/BCIP. Embryos were then vibratome sectioned (50 μm).

## Competing interests

The author(s) declares that there are no competing interests.

## Authors' contributions

JPD, ML, MR and MP conducted most of the experimental work. KH carried out animal cap assays and part of ectopic overexpression experiments. KP performed *in situ *hybridizations. SA generated *Ptf1a *expression vectors. MP and TP conceived the study. ML and MP designed and coordinated the work and wrote the manuscript. JPD, KH and TP contributed to critical reading of the manuscript and all authors read and approved its final version.
